# Regulation of programmed cell death by Brd4

**DOI:** 10.1038/s41419-022-05505-1

**Published:** 2022-12-20

**Authors:** Jinfeng Hu, Dun Pan, Guo Li, Kunqi Chen, Xiangming Hu

**Affiliations:** 1 Fujian Key Laboratory of Translational Research in Cancer and Neurodegenerative Diseases, School of Basic Medical Sciences, Fujian Medical University, Fuzhou, 350122 China; 2 Department of Gastrointestinal Surgery, The First Affiliated Hospital of Fujian Medical University, Fuzhou, 350005 China; 3 Key Laboratory of Gastrointestinal Cancer (Ministry of Education), School of Basic Medical Sciences, Fujian Medical University, Fuzhou, 350122 China; 4 Present Address: Fujian Key Laboratory of Translational Research in Cancer and Neurodegenerative Diseases, School of Basic Medical Sciences, Fujian Medical University, Fuzhou, 350122 China

**Keywords:** Cell death, Epigenetics

## Abstract

Epigenetic factor Brd4 has emerged as a key regulator of cancer cell proliferation. Targeted inhibition of Brd4 suppresses growth and induces apoptosis of various cancer cells. In addition to apoptosis, Brd4 has also been shown to regulate several other forms of programmed cell death (PCD), including autophagy, necroptosis, pyroptosis, and ferroptosis, with different biological outcomes. PCD plays key roles in development and tissue homeostasis by eliminating unnecessary or detrimental cells. Dysregulation of PCD is associated with various human diseases, including cancer, neurodegenerative and infectious diseases. In this review, we discussed some recent findings on how Brd4 actively regulates different forms of PCD and the therapeutic potentials of targeting Brd4 in PCD-related human diseases. A better understanding of PCD regulation would provide not only new insights into pathophysiological functions of PCD but also provide new avenues for therapy by targeting Brd4-regulated PCD.

## Facts

- Brd4 regulates several programmed cell death (PCD) pathways.
- Brd4 is frequently overexpressed or dysregulated in a wide range of human cancers.
- Brd4 is well known for providing cancer cells with a survival advantage by suppressing apoptosis, however new anti-apoptotic and pro-apoptotic members transcriptionally regulated by Brd4 are constantly emerging.
- Small-molecule inhibitors of Brd4 exhibit promises in therapeutic intervention of cancer by inducing cancer cells to die in different PCD pathways, including apoptosis, autophagy, pyroptosis, and ferroptosis.
- Brd4 inhibitors have also been studied in animal models for the treatment of various PCD-related diseases, including cardiovascular, autoimmune and infectious diseases.

## Open questions

- How does Brd4 have both pro-apoptotic and anti-apoptotic potentialities?
- Is there a non-transcriptional role of Brd4 in the regulation of PCD?
- Brd4 has two main isoforms, Brd4 long (Brd4-L) and Brd4 short (Brd4-S), which show different interaction patterns and distinct dynamics of transcriptional activity. Whether the two Brd4 isoforms have opposing activities in regulating PCD?
- Current Brd4 inhibitors all bind to the bromodomain structure and show poor selectivity for individual BET family members. How to overcome the drawbacks of Brd4 inhibitors to avoid off-target effects?

## Introduction

The epigenetic reader Brd4 (bromodomain-containing protein 4), a member of the bromodomains and extraterminal (BET) protein family, is characterized by two conserved N-terminal bromodomains (BD1 and BD2) and an extraterminal (ET) domain [1]. Brd4 was originally identified as a transcription regulator for RNA polymerase II-mediated gene expression. Through binding to acetylated histone and non-histone proteins via its bromodomains, Brd4 recruits different chromatin and transcriptional regulators to control gene expression. Genome wide studies indicate that Brd4 is present at a significant proportion of active promoter and enhancer regions, including super-enhancers [2]. Brd4 recruits positive transcription elongation factor b (P-TEFb) to the promoter-proximal regions for phosphorylation of RNA polymerase II, leading to the start of transcription elongation [3]. Binding to hyperacetylated chromatin regions, Brd4 recruits the mediator complex and chromatin modifiers to facilitate the assembly of a large protein complex, which forms a bridge between enhancer and promoter, promoting and stabilizing the binding of RNA polymerase II [4, 5]. Consistent with its general role in transcriptional regulation, Brd4 plays a crucial role in the control of embryonic development, cell growth and division, metabolic process and immune response [6]. However, dysregulation of Brd4 has been linked to variety of human diseases, including cancer, immune disorders, and metabolic diseases [2, 7, 8]. For example, Brd4 is found to be overexpressed in many kinds of cancers and is involved in the initiation and progression of cancer by providing survival signals to cancer cells [9–11]. Inhibition of Brd4 reduces cancer cell viability in vitro and suppresses tumor growth in vivo, by inducing cell apoptosis, autophagy, pyroptosis or ferroptosis in different contexts, indicating a critical role of Brd4 in the regulation of program cell death (PCD) [12–16].

PCD programs are required for normal cell turnover and tissue homeostasis by eliminating the damaged, dysfunctional, or potentially harmful cells, including cancer cells and cells infected with pathogens [17–19]. Dysregulation of these normal cell death processes leads to a variety of human diseases, including cancer, neurodegenerative, cardiovascular, autoimmune and infectious diseases [20, 21]. Brd4 possess diverse biological functions largely by its ability to control gene expression in various biological processes [22–25]. The tightly regulated expression of Brd4 is critical for the maintenance of the cell cycle progression in normal tissues, whereas overexpression or abnormal activation of Brd4 is a significant hallmark of the hyperproliferative cells associated with numerous hematological malignancies and solid tumors [26–28]. Brd4 is implicated in many of the pathological processes resulting from dysregulated PCD [15, 29–31]. Accumulating evidence indicates that the pathophysiological roles of Brd4 might result from its ability to control various PCD programs in different biological and pathological settings. In this review, we will focus on the mechanisms by which Brd4 regulates different forms of PCD and discuss the therapeutic potentials by targeting Brd4-mediated PCD.

### Brd4 and apoptosis

**Fig. 1:**
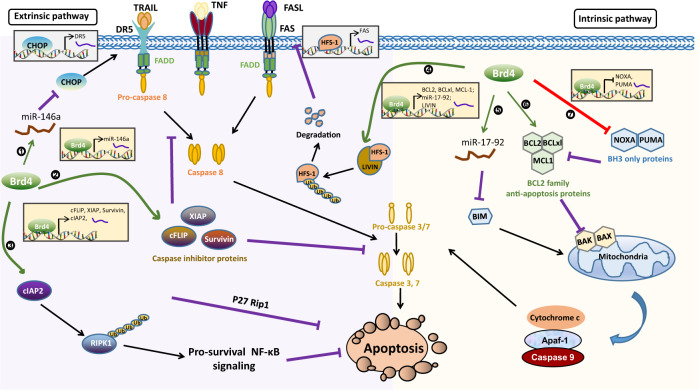
Schematic model for the transcriptional regulation of Brd4 in cellular apoptosis. Brd4 can inhibit apoptosis through both intrinsic (mitochondrial) and extrinsic (death receptor-mediated) mechanisms by regulating the expression of many key components of these pathways. Brd4 directly or indirectly either upregulates the expression of genes coding anti-apoptotic proteins (BCL-2, BCL-xL, MCL-1, LIVIN, cFLIP, XIAP, Survivin and cIAP2) or downregulates the expression of genes coding pro-apoptotic proteins (NOXA, PUMA, BIM and CHOP).

Apoptosis, known as type I programmed cell death, is the primary cell death model involved in development and tissue homeostasis, whereas its dysregulation underlies an enormous number of pathological conditions [32]. Apoptosis is characterized by cellular shrinking, nuclear condensation and fragmentation, dynamic membrane blebbing and intracellular substrate cleavage by proteolysis [33]. Through activating caspase-family proteases involved in both the intrinsic (mitochondria-mediated) and extrinsic (death receptor-mediated) pathways, apoptosis eliminates heavily damaged cells or unwanted cells during embryonic and adult development [32]. Recent studies indicate that both intrinsic and extrinsic apoptosis pathways can be directly or indirectly regulated by Brd4 (Fig. 1 and Table 1).

**Table 1:** Brd4 mediated programmed cell death Pathways.

Target substrates	Cooperates with transcriptional regulator		Regulation by Brd4	Signaling pathway	Cellular type	BETi	Ref.
**Apoptosis**							
**(1) Pro-apoptotic proteins**							
NOXA		P53	Down, mRNA, direct	P53	Acute myeloid leukemia	CPI203, JQ1	[41, 42]
PUMA		P53	Down, mRNA, direct	P53	Acute myeloid leukemia	CPI203, JQ1	[41, 42]
BIM		miR17–92	Down, mRNA, indirect	BCL-2	Eµ-Myc lymphoma, MM.1 S, HL-60 and MV4;11	JQ1	[44, 45]
CHOP		miR-146a	Down, mRNA, indirect	DR5	Colorectal cancer cells	JQ1, I-BET151, I-BET762, OTX015	[48, 49]
FoxO4		N/A	Up, mRNA, indirect	PI3K/AKT	Renal epithelial cells	JQ1	[68]
**(2) Anti-apoptotic proteins**							
BCL-2		N/A	Up, mRNA, direct	BCL-2	Mixed-lineage leukemia, T cell acute lymphoblastic leukemia	JQ1, I-BET151	[29, 38]
BCL-XL		MED1	Up, mRNA, direct	BCL-2	Multiple myeloma	JQ1	[4]
MCL-1		MED1	Up, mRNA, direct and Protein stability	BCL-2	Multiple myeloma	JQ1, ZBC260, dBET	[4, 40]
LIVIN		N/A	Up, mRNA, direct	FAS	Refractory lymphoma cells	JQ1, ARV-825	[51]
cFLIP		N/A	Up, mRNA, direct	Caspase 8	Non-small cell lung cancer	JQ1, I-BET762, OTX015	[59]
XIAP		N/A	Up, mRNA, direct	Caspase 8, 3, 7	Non-small cell lung cancer	JQ1, I-BET762, OTX015	[59]
Survivin		NF-κB	Up, mRNA, direct	Caspase 3, 7	Glioblastoma cells	JQ1, I-BET151, I-BET762	[58]
cIAP2		NF-κB	Up, mRNA, direct	NF-κB	Gastric cancer cells	JQ1	[57]
Caveolin-1		N/A	Down, Protein	AKT	cardiomyocytes	JQ1	[69]
**Autophagy**							
Autophagosome formation genes, e.g., BECN1, AP1LC3B		G9a	Down, mRNA, direct	mTORC	Pancreatic adenocarcinoma cells	JQ1, I-BET762, OTX015	[13]
Autophagolysosome formation genes, e.g., PLEKHM1, TECPR1		G9a	Down, mRNA, direct	mTORC	Pancreatic adenocarcinoma cells	JQ1, I-BET762, OTX015	[13]
Lysosome genes, e.g., GNS, SGSH, TPP1		G9a	Down, mRNA, direct	mTORC	Pancreatic adenocarcinoma cells	JQ1, I-BET762, OTX015	[13]
AMPK		N/A	Suppress its activity	mTORC	Breast cancer cells	9 f (FL-411)	[14]
**Necroptosis**							
MLKL		IRF1/ P-TEFb	Up, mRNA, direct	RIPK1/RIPK3/ MLKL	HT29, L929	JQ1, I-BET151, I-BET762	[98]
**Pyroptosis**							
NAIP2, 5, 6		IRF8-PU.1	Up, mRNA, direct	NLRC4	BMDM	N/A	[31]
NAIP1, NLRC4		N/A	Up, mRNA, indirect	NLRC4	BMDM	N/A	[31]
pro-IL-18		N/A	Up, mRNA, direct	NLRC4	BMDM	N/A	[31]
Cleaved IL-1β and Casp-1		N/A	Up, Protein	NLRP3/caspase11	BMDM	N/A	[31]
Cleaved IL-1β and Casp-1		N/A	Up, Protein	Pyrin	BMDM	N/A	[31]
NLRP3		NF-κB	Down, mRNA, direct	NLRP3/caspase1	Renal cell carcinoma	JQ1	[15]
NLRP3		N/A	Up, Protein	NLRP3/caspase1	Glial cells, colon or liver tissues	JQ1	[111, 112, 116]
**Ferroptosis**							
FTH1		N/A	Up, protein stability	Ferritinophagy	Breast cancer, lung squamous cell carcinoma	JQ1	[16]
GPX4		N/A	Up, mRNA, indirect	Ferritinophagy	Breast cancer, lung squamous cell carcinoma	JQ1	[16]
SLC7A11		N/A	Up, mRNA, indirect	Ferritinophagy	Breast cancer, lung squamous cell carcinoma	JQ1	[16]
SLC3A2		N/A	Up, mNRA, direct	Ferritinophagy	Breast cancer, lung squamous cell carcinoma	JQ1	[16]

Intrinsic apoptosis stimuli, such as DNA damage and growth factor withdrawal, trigger pro-apoptotic members of B-cell lymphoma 2 (Bcl-2) family-mediated mitochondrial outer membrane permeabilization (MOMP), which leads to the release of cytochrome c from mitochondria to the cytoplasm, where it binds to apoptotic protease activating factor 1 (APAF-1), leading to the activation of Caspase-9, and the subsequent activation of Caspase-3 and Caspase-7 [33, 34]. The pro-survival and pro-apoptotic *BCL*-*2* family proteins determine the commitment of cells to apoptosis, making the life-or-death decision for cells [35]. In many hematological malignancies and solid tumors, the expression of pro-survival BCL-2 is directly regulated by Brd4 [29, 36, 37]. Brd4 binds to the promoter and super-enhancer region of *BCL-2* gene to facilitate BCL-2 expression [29, 38]. Inhibition of Brd4 by small molecules JQ1 or I-BET151 downregulates BCL-2 expression and induces growth arrest and apoptosis in mixed lineage leukemia (MLL) or T cell acute lymphoblastic leukemia (T-ALL) [29, 32, 39]. Two other pro-survival BCL-2 family proteins, BCL-2-related gene long isoform (BCL-xL) and myeloid cell leukemia-1 (MCL-1), are also targets of Brd4. Brd4 is recruited to the super-enhancers that are co-occupied by Mediator subunit MED1 to drive the synthesis of *BCL2L1* and *MCL1* mRNA [4]. It is interesting to note that BET degrader ZBC260 reduces MCL-1 protein levels through enhancing its proteasomal degradation in addition to suppression of its transcription [40]. Knocking down of Brd2 and Brd4 together facilitates MCL-1 degradation as effectively as ZBC260, although the exact mechanisms remain unclear [40], indicating that Brd4 may regulate MCL-1 expression at the levels of transcription and protein stability.

The BH3-only proteins of the BCL-2 family, which convey various cytotoxic signals, are also regulated by Brd4. Rather than a transcriptional co-activator, Brd4 functions as a transcriptional repressor of BH-3 only proteins, such as P53 upregulated modulator of apoptosis (PUMA) and phorbol-12-myristate-13-acetate-induced protein 1 (NOXA) [41, 42]. In one study, Brd4 is shown to bind to the promoter region of *PUMA* and *NOXA* and suppress their transcription although the underlying mechanisms remain undetermined [41]. Brd4 is able to suppresses gene expression by the recruitment of histone methyltransferase G9a [13, 43]. Conceivably, the interaction of Brd4 and G9a might be involved in Brd4-mediated repression of these pro-apoptotic genes. In a different study, Brd4 regulates the expression of BH3-only protein BIM via miR17–92, a key post-transcriptional repressor of BIM expression [44, 45]. Brd4 binds to the promoter of miR17–92 to facilitate its expression, which suppresses the transcription of BIM, leading to the inhibition of apoptosis [44]. Therefore, Brd4 can suppress the expression of these pro-apoptotic genes at the transcriptional and post-transcriptional levels.

In addition to the intrinsic pathway, Brd4 also regulates the extrinsic pathway of apoptosis. The extrinsic apoptotic pathway is initiated by activation of cell membrane proteins known as death receptors (DRs), which include the TNF receptor 1 (TNFR1, also known as DR1), FAS (DR2, its ligand is FasL), and the TNF-related apoptosis-inducing ligand (TRAIL) receptors DR4 and DR5. Upon ligands binding, death receptors recruit adapter protein FAS-associated protein with death domain (FADD) and initiator caspases, such as caspase 8 and 10, to form the death-inducing signaling complex (DISC). Under normal situation, Caspase-8 and 10 are inactive with their interactions with cellular FLICE-like inhibitory protein (cFLIP) [46]. When cFLIP is absent, Caspase-8 and 10 are activated, triggering the activation of Caspase-3 and 7, and cell apoptosis [47].

In colorectal cancer cells, DR5 and Endoplasmic reticulum (ER) stress response genes, including C/EBP homologous protein (CHOP), are significantly induced in response to Brd4 depletion and BET inhibitors (BETi) treatment [48]. Furthermore, CHOP is found to bind to the DR5 promoter upon JQ1 treatment and knockdown of CHOP can abrogate the induction of DR5 by JQ1 [48]. The regulation mechanism of Brd4-suppressed CHOP expression is unclear. One possibility is that Brd4 might down-regulate the expression of CHOP via miR-146a, which is able to target CHOP [49]. Interestingly, the expression of miR-146a is driven by Brd4 and NF-κB co-occupied super-enhancers [50]. Moreover, apoptosis of colorectal cancer cells induced by Brd4 knockdown or inhibition in combination with chemotherapeutic drugs, such as 5-fluorouracil (5-FU) or oxaliplatin, is markedly suppressed with DR5 genetic deletion [48].

In lymphoma and nasopharyngeal carcinoma cells, FAS abundance is negatively correlated with the expression of LIVIN [51, 52]. LIVIN binds to heat shock factor-1 (HSF-1), the transcription factor of FAS, leading to increased ubiquitination and accelerated degradation of HSF-1 [52]. The cells expressing LIVIN are significantly resistant to FASL-induced apoptosis [51]. BET inhibitor or Brd4 degrader decreases LIVIN expression in a concentration-dependent manner [51]. Consistently, BET family proteins, such as Brd4 and Brd2, enhance transcription of *BIRC7* (encodes LIVIN) via binding to its promoter/enhancer [51]. By regulating the expression of LIVIN via HSF-1, Brd4 indirectly regulates the expression of FAS and extrinsic pathway of apoptosis.

LIVIN, also known as melanoma inhibitor of apoptosis protein (ML-IAP), is a member of the inhibitor of apoptosis proteins (IAPs) family that confer protection from intrinsic or extrinsic apoptosis by inhibiting caspase activation [53]. Generally, IAP family proteins characterized by a novel domain termed the baculoviral IAP repeat (BIR), comprise 8 members (BIRC1–8), including NAIP/*BIRC1*, cIAP1/*BIRC2*, cIAP2/*BIRC3*, XIAP/*BIRC4*, Survivin/*BIRC5*, Apollon/*BIRC6*, ML-IAP/*BIRC7* and ILP2/*BIRC8* [53]. The activation or activity of both initiator and executioner caspases can be regulated by IAPs [53].

Upregulation or dysfunction of XIAP, cIAP1 and cIAP2 is required to maintain apoptosis resistance in various types of cancer cells [54–56]. It has been shown that *H. pylori* infection-induced apoptosis resistance in gastric cancer cells resulted from Brd4-mediated induction of cIAP2 [57]. *H. pylori* stimulates the recruitment of Brd4, likely through NF-κB, to the enhancer of *BIRC3* and promotes its enhancer RNA (eRNA) and mRNA synthesis [57]. Inhibition of Brd4 diminishes the expression of cIAP2 and the apoptosis resistance induced by *H. pylori* infection.

Other caspase inhibitory proteins regulated by Brd4 include Survivin [58], XIAP and cFLIP [59]. Brd4 has been shown to associate with NF-κB on the promoter of *BIRC5* (gene for Survivin) to facilitate mRNA expression of Survivin in glioblastoma cells with IL-6 stimulation [58]. Brd4 inhibition abrogated an the constitutively active mutant form of the EGFR receptor (EGFRvIII)-induced transcription expression of *BIRC5* and eliminated IL-6-mediated therapy resistance in glioblastoma [58]. Although Brd4 has been demonstrated to occupy at the promoter of *BIRC4* or *cFlip* [59], the detailed mechanisms remain largely unknown. NF-κB has been shown to mediate the recruitment of Brd4 and P-TEFb to most, but not all NF-κB target genes, which in turn facilitates transcriptional elongation by RNA Polymerase II [60–62]. Since *BIRC4* or *cFlip* promoter can be transactivated by NF-κB [63, 64], it is possible that NF-κB recruits Brd4/P-TEFb complex to their promoters to stimulate the expression.

Brd4 inhibition has been demonstrated to induce apoptosis in many different types of cancer cells [12, 65–67]. However, recent studies have also demonstrated that Brd4 inhibition alleviates apoptosis of non-transformed cells under certain pathological conditions [68, 69]. For example, Renal ischemia/reperfusion (IR) injury leads to Forkhead box O4 (FOXO4)-mediated generation of ROS, which triggers cellular apoptosis by increasing the expression of pro-apoptotic genes including Bcl-6, Bim, and Bax [70]. This apoptosis is abolished by JQ1 treatment or Brd4 inhibition [68]. Mechanistically, Brd4 inhibition downregulates FoxO4 expression through activating upstream PI3K/AKT pathway and then transcriptionally decreases FoxO4 promoter activity, which finally suppresses oxidative stress-induced apoptosis [68]. Another recent study has reported that inhibition of Brd4 reduces apoptosis of cardiomyocytes under hyperglycemia condition by restoring the Caveolin-1/AKT signal [69]. Caveolin-1, a scaffolding protein within caveolar membranes, which affects the apoptotic process by regulating the expression and activation of downstream proteins (such as Hsp90, ROS and onco-proteins) or apoptotic pathway proteins (such as Bcl-2, Bax and caspase-3) [71]. Caveolin-1 deficient heart is vulnerable to ischemic cardiomyocyte apoptosis, concomitant with increased Bax/Bcl-2 ratio and decreased p-AKT activity [72]. In high-glucose stimulated cardiomyocytes and diabetic myocardium, Caveolin-1 expression is decreased but reversed by JQ1, indicating that the expression of Caveolin-1 could regulated by Brd4 [69].

### Brd4 and autophagy

Autophagy is a cytoprotective process that cells degrade and recycle misfolded proteins, damaged organelles, intracellular pathogens, and other abnormal components [73, 74]. A moderate autophagic response serves as a survival mechanism that contributes to maintaining cellular homeostasis under normal or stress conditions, such as hypoxia, oxidative stress, nutrient deprivation and microbial invasion [75]. Dysfunction of autophagy is detrimental to the cells and is associated with a number of human diseases, including aging, cancer, neurodegeneration and microbial infection [76].

A key morphological feature of autophagy is autophagosome formation, which is a multi-phase process that can be mainly divided into four stages, including initiation, elongation, maturation and fusion [77]. The autophagy process starts at the birth of the phagophore, a small double layer membrane, which begins to wrap the targeted organelles or components to be eliminated. The double-membrane elongates and closes to form the autophagosome, a double-membrane vesicle, which subsequently fuses with the lysosome to form the autophagolysosome, in which the hydrolytic lysosomal enzymes catabolize the autophagosomal contents into metabolic substrates [78]. As a non-apoptotic form of PCD, autophagy is a complex process involving numerous upstream signaling pathways and >40 autophagy-related (ATG) proteins [79, 80].

**Fig. 2:**
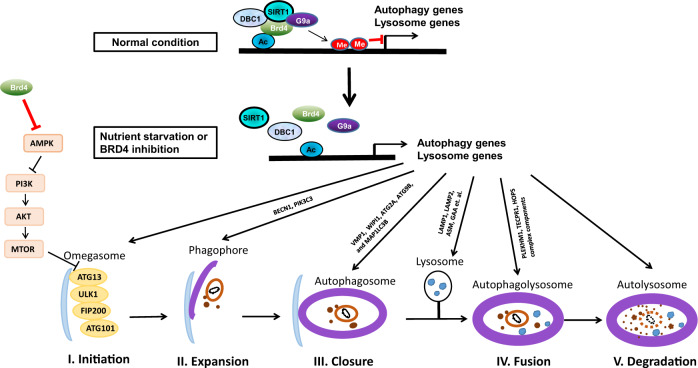
Model of Brd4 modulating autophagy. In normal condition, Brd4 occupies the promoter region with DBC1, SIRT1 and G9a and represses the expression of genes involving in the five phases of autophagy. In the case of starvation or Brd4 inhibition, Brd4 is displaced from chromatin instigating autophagy and lysosome-related genes activation. In addition, Brd4 can prevent autophagy via interacting with AMPK and then activating PI3K-AKT-MTOR pathway.

New research has shown that knockdown or inhibition of Brd4 induces autophagy by increasing the expression of several autophagy and lysosomal genes [13]. Different from the its common role as a positive transcription factor, Brd4 negatively regulates the autophagy-related genes (Fig. 2), including genes involved in phagophore/autophagosome formation (*BECN1*, *PIK3C3*, *ATG2A*, *ATG9B*, *MAP1LC3B* and *MAP1LC3C*) and autophagosome/lysosome fusion (*PLEKHM1*, *TECPR1*, and genes coding components of the homotypic fusion and vacuole protein sorting (HOPS) complex) [13]. In addition, Brd4 silencing upregulates lysosome genes involved in proteolysis, glycan degradation, and lysosome biogenesis [13]. Mechanistically, Brd4 binds to the promoters of autophagy-related genes via histone H4K16 acetylation and recruits the histone methyltransferase G9a that dimethylates H3K9, resulting in the transcriptional repression of autophagy-related genes [13, 81]. However, G9a knockdown largely, but not completely, abolishes autophagy suppression by Brd4, which indicates that other mechanism(s) might modulate the autophagy suppression by Brd4. JQ1 treatment dissociates Brd4 from the promoter regions and promotes autophagy-related gene expression and autophagy activity [13].

Of note, Brd4 repression contributes to autophagy under stimulus conditions, such as nutrient deprivation, hypoxia, protein aggregates, and oncogenic Ras mutant, but is not involved in the autophagic removal of mitochondria or bacteria [13]. Another study has reported that JQ1 or the PI3K/mTOR/Brd4 inhibitor SF2523 can decrease HIV replication in macrophages via autophagic degradation of intracellular HIV [30]. Therefore, Brd4 inhibition promotes certain, but not all types of autophagy.

### Brd4 and necroptosis

Necroptosis is a programmed form of necrotic cell death and can be triggered by various stimuli, including TNF, Toll-like receptor agonizts, second mitochondrial activator of caspases (Smac) mimetics, DNA-damaging agents or vial/bacterial infection [82–87].

Most of what we know about necroptosis comes from studies of TNF-induced necroptosis, which requires the involvement of the serine/threonine protein kinases receptor-interacting proteins 1 and 3, (RIP1 and RIP3) and the effector protein mixed-lineage kinase domain like (MLKL) [84, 88–90]. Upon TNF stimulation, RIPK1 and TNF receptor-associated death domain (TRADD) are recruited to the TNF receptor (TNFR) at the plasma membranes, leading to the assembly of complex I [91]. At the complex I, RIPK1 is inactive due to its ubiquitylation by cIAP1/2 and other E3 ubiquitin ligases. Inactive RIP1 recruits the transforming growth factor β-activated kinase 1 (TAK1) and IκB kinase (IKK) complexes, leading to the activation of NF-κB and mitogen-activated protein kinases (MAPKs) [92, 93]. In response to a death signal, RIPK1 is deubiquitylated by cylindromatosis (CYLD) and A20, resulting in its dissociation from TNFR and interaction with FADD and caspase 8 to form Complex IIa [82]. If caspase 8 is inactive or absent, pro-apoptotic complex IIa convert into a pro-necroptotic complex IIb, with kinase-active RIPK1 recruiting and activating RIPK3 [82, 94]. The RIPK1/RIPK3 complex binds to and phosphorylates MLKL, thus forming a complex called the necrosome [90, 95]. Phosphorylated MLKL oligomerizes and translocates from the cytoplasm into the plasma membranes, where it disrupts membrane integrity to execute necroptotic cell death [96, 97].

**Fig. 3:**
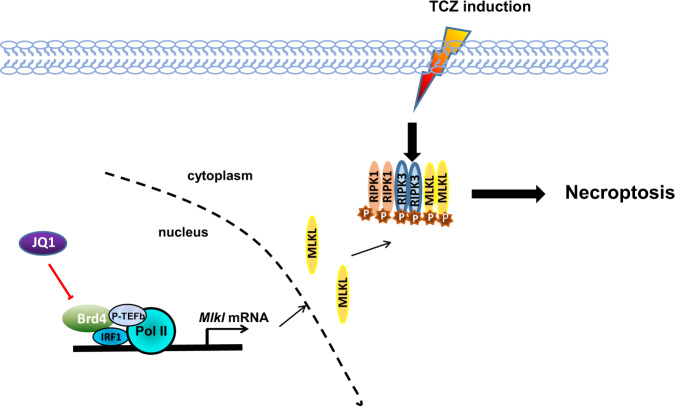
The necroptosis mechanism regulated by Brd4. Under the signal of necroptosis, Brd4 is recruited to the promoter of MLKL and forms a transcription complex with IRF1, P-TEFb, and RNA polymerase II to regulate the expression of MLKL, which leads to necroptosis. BET inhibitors such as JQ1 interferes with the transcription complex formation.

Although the fundamental axis of RIPK1-RIPK3-MLKL has been identified, other regulatory factors that contribute to necroptosis remains largely unknown [98]. A recent study identified Brd4 as a new epigenetic factor of necroptosis via cell-based small molecule screening [98]. Brd4 regulates the expression of MLKL by forming a transcription complex with IRF1, P-TEFb, and RNA polymerase II on the promoter region of *MLKL* gene (Fig. 3). Knockdown or inhibition of Brd4 by JQ1 interferes with the formation of this transcription complex, leading to decreased MLKL expression, thereby protecting cells from necroptosis [98]. Another recent study has reported that JQ1 alleviates listeria monocytogenes-induced acute liver injury by suppressing necroptosis via inhibiting RIPK1 over-expression, but the molecular mechanism of inhibiting RIPK1 by JQ1 remains undetermined [99].

### Brd4 and pyroptosis

Pyroptosis is a pro-inflammatory type of programmed cell death characterized by inflammasome activation, associated with the release of intracellular pro-inflammatory contents, including IL-1β, IL-18 and high mobility group protein 1 (HMGB1) [100, 101]. In the canonical inflammasome pathway which requires the conversion of pro-caspase-1 into catalytically active caspase-1, pathogen-associated molecular patterns (PAMPs) or danger-associated molecular patterns (DAMPs) activate their respective sensors, which include the nucleotide-binding oligomerization domain (NOD)-like receptor (NLR) family, the absent in melanoma 2 (AIM2), and Pyrin and PYHIN protein family [102]. Upon detection of specific stimuli, the senor proteins recruit the inflammasome adapter ASC (apoptosis-associated speck-like proteins) to form a large protein complex termed ASC specks to initiate caspase-1 activation [103], which in turn cleaves pro-IL-1β and pro-IL-18 for the maturation of IL-1b and IL-18 [104]. Active caspase-1 also cleaves gasdermin D (GSDMD), leaving cleaved N-terminal portion of GSDMD forming pores on plasma membrane, allowing the release of mature IL-1β and IL-18 and induce a form of cell death called ‘pyroptosis’ [105, 106]. So far, at least 5 different kinds of inflammasome have been identified based on the sensor proteins: NLRP1, NLRP3, NLRC4, AIM2 and pyrin [107]. Several of these inflammasome activation are regulated by Brd4.

NLRP3 inflammasome, the most well-characterized inflammasome, could be activated by a multitude of infectious and sterile stimuli, including fungal, bacterial, and viral pathogens, as well as ATP, reactive oxygen species, ionic flux, and mitochondrial dysfunction [108, 109]. Brd4 has been shown to be involved in the activation of NLRP3 inflammasome in different pathophysiological conditions. Inhibition of Brd4 by JQ1 or siRNA suppressed NLRP3 inflammasome activation and pyroptosis in neural cells, resulting in analgesic and anti-inflammatory effects against inflammatory pain [110]. Brd4 suppression has also been shown to alleviate cerebral ischemia-induced brain injury by inhibiting NLRP3-medaited inflammatory response and pyroptosis in glial cells [111]. In another study, Brd4 inhibition blocked NLRP3 inflammasome activation in endotoxemia colon [112]. In most cases, Brd4 function as a positive regulator of NLRP3 inflammasome activation by facilitating NF-κB-dependent transcription of NLRP3, the priming step for NLRP3 inflammasome activation [110–112]. Intriguingly, Brd4 could also serve as a negative regulator of NLRP3 inflammasome activation. Inhibition of Brd4 activated NLRP3 inflammasome-induced pyroptosis and prevented proliferation and epithelial–mesenchymal transition in renal cell carcinoma [15]. It appears that Brd4 regulates NLRP3 inflammasome activation on a stimulus-specific and cell type-specific manner. Supporting this, Brd4 is not involved in nigericin-induced NLRP3 inflammasome activation in macrophages [31]. However, Brd4 regulates caspase-11–mediated noncanonical NLRP3 inflammasome activation because intracellular LPS-activated cleavage of pro-caspase-1 and pro-IL-1β, secretion of IL-1 β, and pyroptosis were decreased in *Brd4*-deficient BMDMs [31].

NLRC4 inflammasome activation is also regulated by Brd4. NLRC4 inflammasome is activated in response to bacterial type III secretion system (T3SS) components and flagellin, which are detected by the NLR family of apoptosis inhibitory proteins (NAIPs), such as NAIP1/2 and NAIP5/6 [113]. NLRC4 inflammasome components, including NAIPs and NLRC4, are regulated by Brd4. Brd4 forms a complex with IRF8 and PU.1 on the promoters of *Naip2, 5, 6* to facilitate their expression in macrophages [31]. Both IRF8 and PU.1 are essential for the complex formation and Naips transcription, since mutation of either IRF8 or PU.1 binding motif disrupts the complex formation and IRF8/PU.1-medaited transcription of Naips [31]. In addition to Naips, Nlrc4 is also transcriptionally regulated by Brd4 since the expression of Nlrc4 is similarly decreased in *Brd4*-deficient BMDMs [31]. However, the regulation mechanism could be different. Brd4 and IRF8 was not enriched on the promoter of Nlrc4. Instead, Brd4 and IRF8 were found to be enriched in the same region within intron 5 of *Nlrc4* [31], indicating that Brd4 might cooperate with IRF8 to regulate *Nlrc4* transcription through this unique intronic region. Brd4-mediated optimal NLRC4 inflammasome activation is critical in host innate immunity against *Salmonella* infections since *Brd4*-deficient BMDMs displayed reduced IL-1β and IL-18 expression and *Brd4-*conditional knockout mice exhibited increased bacterial loads in various tissues and mortality upon *S. typhimurium* infection [31].

**Fig. 4:**
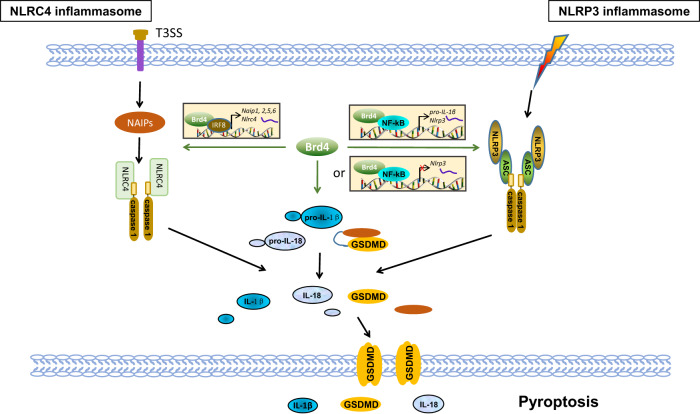
Schematic model for the regulation of Brd4 in pyroptosis. Brd4 transcriptionally upregulates the expression of pro-IL-1β, NAIP1, 2, 5, 6 and NLRC4. Brd4 upregulates or downregulates NLRP3 expression depending on the context.

AIM2 inflammasome is activated by cytoplasmic dsDNA [114], whereas Pyrin inflammasome senses bacterial toxin-induced modifications of Rho GTPases [115]. While AIM2 inflammasome activation remains intact in *Brd4*-deficent BMDMs, Pyrin inflammasome activation triggered by clostridium difficile toxin B is decreased in *Brd4*-deficent BMDMs [31]. The regulation of pyrin inflammasome by Brd4 is likely at the transcription level since pyrin mRNA is significantly down-regulated in *Brd4*-deficent BMDMs [31]. Therefore, Brd4 appears to modulate the activation of different forms of inflammasome in response to distinct stimuli and the detailed molecular mechanisms merit further investigation (Fig. 4) [15, 110, 112, 116–119].

### Brd4 and ferroptosis

Ferroptosis is characterized by iron-dependent accumulation of lethal level of lipid reactive oxygen species (ROS) from lipid peroxidation [120]. Ferroptosis has neither the typical morphological features of necrosis, such as plasma membrane rupture and organelle swelling, nor the morphological features of apoptosis, such as plasma membrane blebbing, cytoplasmic shrinkage and nuclear condensation. Also, unlike autophagy, ferroptosis does not have the formation of double-membraned autolysosomes; instead, it manifests primarily as shrinkage of mitochondria with condensed membrane density, reduction or vanishing of mitochondrial cristae, and rupture of outer mitochondrial membrane, which is a process that differs from other modes of cell death [120, 121].

Ferroptosis could be triggered by the accumulation of glutamate, iron or polyunsaturated fatty acids (PUFAs), or by the depletion of intracellular glutathione (GSH) or glutathione peroxidase 4 (GPX4), leading to the accumulation of ROS [122, 123]. Excess levels of ROS induce lipid peroxidation and unrepairable damage of lipid membranes, followed by oxidative cell death [124].

**Fig. 5:**
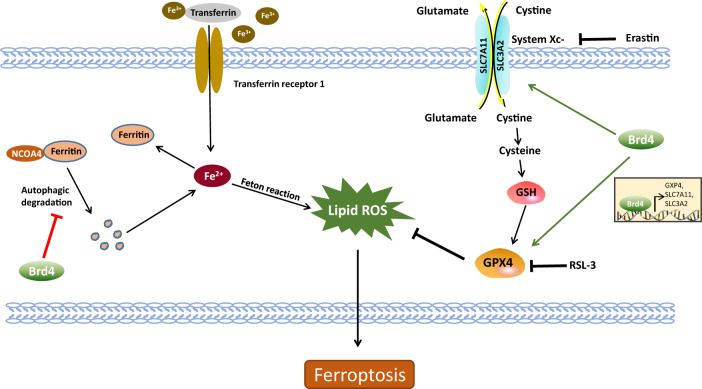
Roles of Brd4 in ferroptosis. Brd4 suppresses ferroptosis by transcriptionally promoting the expression of GAP4, SLC7A11 and SLC3A2 and inhibiting the autophagic degradation of ferritin.

GSH is intracellular antioxidant synthesized from glutamate, cysteine, and glycine. The rate of GSH synthesis is limited by cysteine availability. System Xc- is an amino acid antitransporter that imports extracellular cysteine into cells. Inhibiting the activity of system Xc- by erastin and its analogs, affects the synthesis of GSH by preventing cystine uptake, which finally leads to ferroptosis [120, 125]. GPX4 is a vital suppressor of ferroptosis, which specifically reduces the cytotoxic lipid peroxides (L-OOH) to non-toxic lipid alcohols (L-OH) by utilizing GSH [126]. A recent report has shown that the transcription of *GPX4*, *SLC7A11*, and *SLC3A2* (the latter two genes respectively codes two subunits of System Xc-) are decreased in Brd4 knockdown cells (Fig. 5), however, the specific mechanisms by which Brd4 regulates them need further exploration [16].

Iron metabolism plays key roles in ferroptosis [127]. Iron chelators suppress ROS accumulation and block ferroptosis in vitro and in vivo [120], while excess of heme and non-heme iron can directly induce ferroptosis [128]. Thus, the import, export, storage, utilization and turnover of iron affects the sensitivity of cells to ferroptosis. Ferroptosis can be induced under JQ1 treatment and Brd4 knockdown in various cancer cell lines and mouse tumor xenografts [16]. Moreover, the anticancer effect of JQ1 is enhanced by ferroptosis inducers. JQ1 treatment and Brd4 knockdown increase the intracellular iron levels, leading to ROS accumulation and ultimately ferroptosis via the autophagic degradation of Ferritin Heavy Chain 1 (FTH1), the main intracellular iron-storage protein that binds and sequesters intracellular iron [16].

Aberrant lipid metabolism is also closely associated with ferroptosis. Polyunsaturated fatty acids (PUFAs) are susceptible to lipid peroxidation and are one of the essential elements for ferroptosis [129]. Free PUFAs serve as substrates for synthesis of lipid signal transduction mediators, yet they must be esterified into membrane phospholipids and undergo oxidation to transmit ferroptotic signals [130]. Therefore, ferroptosis can be triggered or blocked by modulating enzymes involved in the biosynthesis or remodeling of PUFA-containing membrane phospholipids. For example, acyl-CoA synthetase long-chain family member 4 (ACSL4) and lysophosphatidylcholine acyltransferase 3 (LPCAT3) are required for the biosynthesis and biosynthesis and remodeling of PUFAs in cellular membranes.

Therefore, Brd4 might be actively involved in ferroptosis by regulating the GSH activity, iron and PUFA metabolism.

### Expression of Brd4 in PCD

Brd4 is ubiquitously expressed in a broad range of somatic cells. Compared to normal cells, Brd4 is highly expressed in various cancer cells (e.g., breast cancer, esophageal cancer, stomach adenocarcinoma, and lung squamous cell carcinoma) [16] and it can protect cancer cells against PCD. When cancer cells undergo PCD, the expression of Brd4 may be further changed. In the case of apoptosis, several miRNAs (e.g., miR-608, miR-204 and miR-29b) promote apoptosis via downregulation of Brd4 [131–133]. Speckle-type POZ protein (SPOP) is an E3 ubiquitin ligase adapter of Brd4 [134]. Depending on tumor type and specific residues affected, SPOP mutations could be activating or inactivating, with opposing effects on BETi sensitivity [48, 134]. SPOP-inactivating mutations in prostate cancer cells stabilize Brd4 and confer resistance to BETi, while SPOP-activating mutations in colorectal cancer cells with reduced Brd4 expression are exquisitely sensitive to BETi and prone to apoptosis [48, 134]. Brd4 is upregulated in colon tissue of mice with endotoxemia and promotes pyroptosis-related acute colon injury, however, the regulatory mechanism controlling Brd4 expression is unclear [112]. A recent study has reported that curcumin degrades Brd4 in foam cells (FCs) by autophagy activation, in turn restoring FCs autophagy and ameliorating inflammation in atherogenesis [135]. It is worthy to note that Brd4 function is not completely determined by its expression, it is also related to its activity controlled by posttranslational modifications, such as phosphorylation [136, 137], methylation [138] and hydroxylation [139], which are unexplored in PCD.

### Therapeutic potential for the PCD-associated diseases by targeting Brd4

As we discussed above, Brd4 plays a role in many of the pathological processes resulting from aberrant PCD, and now there are some achievements in targeting Brd4 for preclinical and clinical studies. According to drug selectivity, small molecule inhibitors of Brd4 are subdivided into pan-bromodomain inhibitors (which do not discriminate between BD1 and BD2 bromodomains), BD1-or BD2-selective inhibitors [140]. JQ1 and I BET were the first-generation BETi discovered in 2010, as acetyl-histone mimetics that competitively bind BD1 and BD2 bromodomains, thus displacing BET proteins from chromatin [141, 142]. JQ1 induces progressive apoptosis of human carcinoma cells depending on Brd4 and exhibits anti-tumor effects [141]. Since then, a large number of BETi (e.g., I-BET151, CPI-203, I-BET762 and OTX015) have been developed, which are derivative classes of JQ1 or I-BET [143]. Those pan-BETi have a broader spectrum of prospects for the treatment of hematologic malignancies (e.g., acute myeloid leukemia, mixed lineage leukemia and T cell acute lymphoblastic leukemia) and solid tumors (e.g., colorectal cancer, renal cell carcinoma, lung cancer and breast cancer), via triggering the apoptosis, pyroptosis and ferroptosis of cancer cells (Table 1). 9 f (FL-411), a novel small-molecule that potently inhibits Brd4-BD1, induces AMPK-modulated autophagic cell death of breast cancer both in vitro and in vivo [14], suggesting that Brd4 inhibition-mediated autophagy also displays a therapeutic potential on breast cancer. Several BETi, such as JQ1, I-BET-151, I-BET-762, OTX015, CPI-0610 and ABBV-744, are currently under preclinical testing and clinical trials against multiple cancers [144–146], as detailed in Table 2.

**Table 2:** BET inhibitors: preclinical and clinical trials in cancer.

BET inhibitor	Selectivity	inhibitor /degrader	Typical range of in vivo dosage (mg/kg)	Cancer types tested in preclincal studies	Current clinical trials
JQ1	Pan-BET	inhibitor	30–50	AML; B-ALL; Burkitt’s lymphoma; DLBCL; colon cancer; ovarian cancer; Hepatocellular cancer; prostate cancer; osteosarcoma; breast cancer; pancreatic cancer; NSCLC; glioblastoma; thyroid cancer	N/A
I-BET-151	Pan-BET	inhibitor	30–50	leukemia; glioblastoma; MM	N/A
I-BET-762	Pan-BET	inhibitor	25–50	Prostate cancer; MM	NMC: Phase I/II (NCT01587703); hematological malignancies: Phase I/II (NCT01943851)
OTX015	Pan-BET	inhibitor	20–50	mature B cell lymphoid tumor; advanced hematological malignancies; TNBC; NMC;	advanced solid tumors: Phase I (NCT02259114 and NCT02698176); hematological malignancies: Phase I (NCT01713582); glioblastoma multiforme: Phase II (NCT02296476)
CPI-0610	Pan-BET	inhibitor	10–60	MM; AML	lymphoma: Phase I (NCT01949883); MM: Phase I (NCT02157636); Peripheral nerve tumors: Phase II (NCT02986919)
BAY1238097	Pan-BET	inhibitor	10–15	MM; AML	solid tumors, lymphoma, NMC, and melanoma: Phase I (NCT02369029)
INCB054329	Pan-BET	inhibitor	25–50	breast cancer; MM	advanced malignancies: Phase I/II (NCT02431260)
ZEN-3694	Pan-BET	inhibitor	N/A	Prostate cancer	Prostate cancer: Phase I (NCT02705469)
RO6870810/ TEN-010	Pan-BET	inhibitor	N/A	N/A	hematological malignancies: Phase I (NCT02308761); solid tumors: Phase I (NCT01987362)
ABBV-744	BD2	inhibitor	4.7	Prostate cancer	AML: Phase I (NCT03360006)
dBET6	Pan-BET	degrader	7.5	T-ALL; breast cancer; glioblastoma;	N/A

(Source: https://clinicaltrials.gov)

*AML* acute myeloid leukemia, *B-ALL* acute B lymphoblastic leukemia, *DLBCL* diuuse large B-cell lymphoma, *NSCLC* non-small cell lung cancer, *MM* multiple myeloma, *TNBC* triple negative breast cancer, *NMC* NUT midline carcinoma, *T-ALL* T-cell acute lymphoblastic leukemia.

Studies have shown that dual BETi and other anticancer inhibitors have synergistic effects in cancer treatment [147]. Compared to respective single inhibition, the combination therapy with MDM2 inhibitors (Nutlin-3) activating p53 and BETi (CPI203) targeting Brd4 presents enhanced anti-acute myeloid leukemia activity, resulting from BETi’s ability to liberate Brd4-mediated repression of p53 target genes, and hence potentiate p53-induced apoptosis [41]. Combination of BETi with BCL-2 family inhibitors can improve cellular response to BCL-2 inhibition. For example, combined treatment of I-BET762 with obatoclax, a small-molecule pan-BCL-2 family inhibitor, overcomes resistance and induces apoptosis in lymphoma cells [39].

The combination of BETi with cancer immunotherapy is of particular interest. Cancer cells can evade T-cell immune responses by trigging T-cell exhaustion [148]. The interaction of programmed cell death receptor- 1 (PD-1) and its ligand (PD-L1) creates an immunoregulatory axis promoting the process of T-cell exhaustion [148], indicating exhausted T cells could be revived by PD-L1 blockade. JQ1 significantly reduced PD-L1 expression by decreasing Brd4 occupancy at the PD-L1 gene locus [149, 150]. Combination therapy with JQ1 and anti-PD-1 antibodies causes synergistic responses in different mouse tumor models (e.g., lymphoma, colorectal cancer and lung cancer) [149, 151, 152]. More recent studies have shown that BETi can also ameliorate chimeric antigen receptors (CAR)-T cell exhaustion by reducing the expression of inhibitory receptors (e.g., PD-1 and T-cell immunoglobulin mucin-domain-containing-3 (Tim-3)), improving metabolic fitness, enhancing proliferative potency, and maintaining properties of stem cell–like and central memory T cells [153–155]. Another recent study has reported that BETi can increase NK cell-activating ligand MICA expression via Brd4 inhibition-modulated downregulation of IRF4, a transcriptional repressor of *MICA*, leading to enhanced NK cell-mediated cytotoxicity against multiple myeloma cells [156]. It is therefore likely that BETi and immunotherapy combinations can lead to remarkable progress of treatment response in preclinical studies. Clinical results of BETi combination therapies are noteworthy.

The second-generation Brd4 inhibitors are synthesized by the technology called proteolysis targeting chimera (PROTAC) [143]. PROTACs are novel compounds that promote targeted protein degradation by binding to an E3-ubiquitin ligase [157]. A typical PROTAC degrader consists of two ligands joined by a linker: one ligand binds to the protein of interest (POI) while the other recruits an E3 ubiquitin ligase [157]. The chemically-induced proximity between the POI and E3 ligase results in proteasomal degradation of the POI [157]. The first-generation BETi can bind the bromodomains of BET proteins and these provide ample choices for BET degrader development. dBET1, the first BET PROTAC, was developed using JQ1 and cereblon as E3 ligase handle resulting in the selective and efficient degradation of BET proteins [158]. In recent years, PROTAC-based BETi have been developed for the degradation of Brd4, such as ARV-825, A1874, dBETs and ZBC260 [40, 51, 159]. Studies have confirmed that PROTACs can effectively induce the degradation of Brd4, which is more effective than JQ1 and other traditional Brd4 inhibitors in inhibiting tumor cell growth and promoting cell apoptosis [37, 160]. Furthermore, since PROTACs can be recycled and used for multiple rounds of target protein degradation, compared to their parent BETi, much lower concentrations of PROTACs can achieve profound therapeutic effects [153, 160]. However, PROTACs are large molecules with poor solubility and cell permeability, limiting their bioavailability [161]. Nanomedicine has raised many expectations to improve the bioavailability of PROTACs. Encapsulating PROTACs for the generation of “nanoPROTACs” would increase the cellular uptake and solubility of PROTACs [161]. Recently, researchers have constructed various nanocarriers incorporating ARV-825 for treatment of melanoma, pancreatic cancer and non-small-cell lung cancer [162–164]. These nanoPROTACs not only significantly increase the solubility and half-life of PROTACs, but also show promising anti-tumor effects at low doses in vitro and in vivo settings [162–164]. Given the impressive preclinical antitumor activity demonstrated by BET PROTACs, BET PROTACs could be potential novel therapeutic approach for cancer treatment.

In addition to cancer treatment, Brd4 inhibitors have been studied in animal models for the treatment of inflammatory diseases, such as sepsis, multiple sclerosis and liver fibrosis that have been reported to be necroptosis-related diseases [98, 142, 165, 166]. The TNFα-driven shock is a widely used necroptosis-related disease model, leading to systemic inflammatory response syndrome (SIRS) [167, 168]. A recent study has reported that JQ1 treatment ameliorates TNFα-driven shock-associated hypothermia and organic injuries, and increases the survival rate through downregulating the necroptosis executor MLKL expression, indicating the promising therapeutic efficacy of Brd4 inhibition in the necroptosis-related diseases [98]. Although there are multiple mechanisms for the therapeutic effect of Brd4 inhibitors, inhibition of necroptosis, but not exclusively, may account in part for the therapeutic effects of Brd4 inhibitors in these models.

## Conclusions

Resistance to apoptosis is one of the hallmarks of cancer cells. Cancer cells can evade apoptosis by upregulating anti-apoptotic proteins or downregulating pro-apoptotic proteins [169]. Brd4 is overexpressed in many cancer cells and assumed to be a negative regulator of apoptosis [16]. Brd4 inhibition promotes apoptosis of cancer cells and has exhibited promising therapeutic effects in cancer treatment [12, 29, 38]. However, some cancer cells are insensitive to BETi-induced apoptosis, leading to treatment failure [170]. Several types of PCD (e.g., necroptosis, pyroptosis and ferroptosis) may serve as a promising secondary cell death process for sensitizing cancer cells to antitumor drugs, particularly for apoptosis-resistant cancer cells [171]. Accumulating research has found that apoptosis is not the only type of death induced by Brd4 inhibition, autophagy, pyroptosis or ferroptosis is also involved in Brd4 inhibition-induced cancer cell death [13, 15, 16]. Ferroptosis inducers eliminate apoptosis-resistant cancer cells and enhance JQ1 therapy [16]. Induction of pyroptosis in cancer cells not only inhibits cell proliferation and promotes cell death, but also exerts benefits on cancer immunotherapies, including immune checkpoint inhibitors and CAR-T therapy [172]. Hence, Brd4 inhibition-induced pyroptosis may enhance anti-tumor immunity. However, since autophagy plays dual roles in tumor suppression and promotion [173], the possibility that Brd4 inhibition-induced autophagy could promote tumor growth should be considered.

Brd4 is an epigenetic reader, mainly controlling gene expression by its interacting with acetylated histone and non-histone proteins on the promoter or enhancer regions of various genes [174, 175]. Consistent with its role as a positive transcription regulator, Brd4 directly or indirectly regulates transcriptional events of programmed cell death related proteins via its complex with P-TEFb or mediators [4, 16, 31, 98]. Notably, Brd4 could also serve as a negative regulator by its interaction with G9a to suppress expression of PCD-related proteins [13]. It is also worth noting that Brd4 has intrinsic kinase and lysine acetyltransferase activities [176, 177]. Whether these activities of Brd4 contribute to PCD remains undetermined. Furthermore, Brd4 exists in a short and a long isoform (Brd4-L and Brd4-S). Brd4-L has an extended C terminus that binds P-TEFb, while Brd4-S lacks this C-terminal extension [178]. The two Brd4 isoforms show different interaction patterns and distinct dynamics of transcriptional activity. Disruption of the balance between Brd4-L and Brd4-S leads to significant biological consequences [179–181]. For example, Brd4-L has a tumor suppressive effect in breast cancer, while Brd4-S exhibits oncogenic properties [179, 182]. Whether the different properties of these two isoforms reflects their distinct abilities to regulate PCD remains another interesting question.

Many Brd4 inhibitors have been developed over the past decade. Most of Brd4 inhibitors entered clinical trials are pan-BET inhibitors that bind BD1 and BD2 of BET family proteins and show poor selectivity for individual BET family members, including Brd2, Brd3, Brd4 and Brdt [183, 184]. Although Brd4 is extensively studied, the exact role of other BET members in diseases and pathological conditions is still far from being fully understood. Side effects of BETi have been observed in clinical trials. For example, toxic effects of BETi in clinical trials includes thrombocytopenia and dysfunctions of the digestive system [185, 186]. Hence, Brd4-specific and even Brd4 isoform-selective inhibitors or PROTAC degraders targeting Brd4 may benefit the treatment of various Brd4-mediated diseases. On the other hand, drug combination is a feasible way to ameliorate the toxicity of BETi and overcome drug tolerance. For example, in cancer treatment, BETi will be used in combination with other anticancer therapies, such as immunotherapy or radiotherapy, which could lead to the induction of a mixed programmed cell death to suppress tumor growth. And beyond that, dual-target inhibitors could be developed by focusing on the synergistic functions of Brd4 [187], providing a new way to overcome the barriers inherent to Brd4 inhibition.

In summary, we have discussed the regulatory mechanisms of Brd4 in five different types of PCDs, and the potential of Brd4 as a therapeutic target in diverse diseases, however the role of Brd4 in other types of cell death, such as paraptosis, efferocytosis and NETosis, needs to be further investigated. Thoroughly understanding the underlying mechanisms of Brd4 in different types of cell death will not only help to clarify the medical conditions applicable to Brd4 inhibitors, but also help to design rational therapeutic regimens to achieve the optimal therapeutic effect.

## Data Availability

All data generated or analyzed during this study are included in this published article.
